# Supplementary material for: Natural Green Spaces, Sensitization to Allergens, and the Role of Gut Microbiota during Infancy
Source: mSystems. 2023 Feb 15;8(2):e01190-22. doi: 10.1128/msystems.01190-22 (PMC10134798; doi:10.1128/msystems.01190-22)
Supplement: TABLE S1 [file msystems.01190-22-s0001.docx]

|  |  | **Atopic sensitizations 1 year** | | | | | | **Food atopic sensitizations 1 year** | | | | | | **Inhalant atopic sensitizations 1 year** | | | | | |
| --- | --- | --- | --- | --- | --- | --- | --- | --- | --- | --- | --- | --- | --- | --- | --- | --- | --- | --- | --- |
|  |  | **≥1** | | | **≥2** | | | **≥1** | | | **≥2** | | | **≥1** | | | **≥2** | | |
|  | **Prevalence Overall  N (%)** | **Yes**  **N (%)** | **No**  **N (%)** | **p** | **Yes**  **N (%)** | **No**  **N (%)** | **p** | **Yes**  **N (%)** | **No**  **N (%)** | **p** | **Yes**  **N (%)** | **No**  **N (%)** | **p** | **Yes**  **N (%)** | **No**  **N (%)** | **p** | **Yes**  **N (%)** | **No**  **N (%)** | **p** |
| Total | 699 (100) | 91 (17.2) | 439 (82.8) |  | 28 (5.3) | 502 (94.7) |  | 73 (13.8) | 457 (86.3) |  | 23 (4.3) | 507 (95.7) |  | 23 (4.3) | 507 (95.7) |  | 1 (0.2) | 529 (99.8) |  |
| Infant sex |  |  |  |  |  |  |  |  |  |  |  |  |  |  |  |  |  |  |  |
| Male | 307 (50.3) | 46 (50.6) | 216 (49.2) | 0.82 | 20 (71.4) | 242 (48.2) | **0.02** | 41 (56.2) | 221 (48.4) | 0.22 | 246 (48.5) | 16 (69.6) | **0.05** | 10 (43.5) | 252 (49.7) | 0.56 | 262 (49.5) | 0 (0.0) | 0.32 |
| Female | 304 (49.8) | 45 (49.5) | 223 (50.8) |  | 8 (28.6) | 260 (51.8) |  | 32 (43.8) | 236 (51.6) |  | 261 (51.5) | 7 (30.4) |  | 13 (56.5) | 255 (50.3) |  | 267 (50.5) | 1 (100.0) |  |
| Missing | 58 |  |  |  |  |  |  |  |  |  |  |  |  |  |  |  |  |  |  |
| Birth weight (grams) |  |  |  |  |  |  |  |  |  |  |  |  |  |  |  |  |  |  |  |
| <3,000 | 119 (19.6) | 20 (21.9) | 81 (18.6) | 0.14 | 7 (25.0) | 94 (18.8) | 0.45 | 16 (21.9) | 85 (18.7) | 0.15 | 5 (21.7) | 96 (19.1) | 0.56 | 6 (26.1) | 95 (18.9) | 0.65 | 0 (0.0) | 101 (19.2) | 0.51 |
| 3000-<3500 | 227 (37.3) | 28 (30.8) | 172 (39.5) |  | 8 (28.6) | 192 (38.5) |  | 22 (30.1) | 178 (39.2) |  | 8 (34.8) | 192 (38.1) |  | 6 (26.1) | 194 (38.5) |  | 0 (0.0) | 200 (38.0) |  |
| 3500-<4000 | 184 (30.3) | 35 (38.5) | 125 (28.7) |  | 11 (39.3) | 149 (29.9) |  | 29 (39.7) | 131 (28.9) |  | 9 (39.1) | 151 (30.0) |  | 8 (34.8) | 152 (30.2) |  | 1 (100.0) | 159 (30.2) |  |
| ≥4000 | 78 (12.8) | 8 (8.8) | 58 (13.3) |  | 2 (7.1) | 64 (12.8) |  | 6 (8.2) | 60 (13.2) |  | 1 (4.4) | 65 (12.9) |  | 3 (13.0) | 63 (12.5) |  | 0 (0.0) | 66 (12.6) |  |
| Missing | 61 |  |  |  |  |  |  |  |  |  |  |  |  |  |  |  |  |  |  |
| Gestational age (weeks) | |  |  |  |  |  |  |  |  |  |  |  |  |  |  |  |  |  |  |
| Preterm (34-36) | 34 (5.6) | 4 (4.4) | 28 (6.4) | 0.26 | 1 (3.6) | 31 (6.2) | **0.03** | 4 (5.5) | 28 (6.2) | 0.35 | 0 (0.0) | 32 (6.4) | **0.04** | 1 (4.4) | 31 (6.2) | 0.76 | 0 (0.0) | 32 (6.1) | 0.37 |
| Early term (37-38) | 145 (23.9) | 29 (31.9) | 97 (22.3) |  | 13 (46.4) | 113 (22.7) |  | 23 (31.5) | 103 (22.7) |  | 11 (47.8) | 115 (22.9) |  | 7 (30.4) | 119 (23.7) |  | 1 (100.0) | 125 (23.8) |  |
| Full term (39-40) | 339 (55.9) | 45 (49.5) | 244 (56.1) |  | 10 (35.7) | 279 (56.0) |  | 34 (46.6) | 255 (56.3) |  | 9 (39.1) | 280 (55.7) |  | 13 (56.5) | 276 (54.9) |  | 0 (0.0) | 289 (55.1) |  |
| Late term (≤41) | 89 (14.7) | 13 (14.3) | 66 (15.2) |  | 4 (14.3) | 75 (15.1) |  | 12 (16.4) | 67 (14.8) |  | 3 (13.0) | 76 (15.1) |  | 2 (8.7) | 77 (15.3) |  | 0 (0.0) | 79 (15.1) |  |
| Missing | 62 |  |  |  |  |  |  |  |  |  |  |  |  |  |  |  |  |  |  |
| Birth mode and IAP |  |  |  |  |  |  |  |  |  |  |  |  |  |  |  |  |  |  |  |
| Vaginal, no IAP | 308 (50.7) | 48 (53.3) | 223 (51.2) | 0.55 | 15 (55.6) | 256 (51.3) | 0.7 | 39 (54.2) | 232 (51.1) | 0.77 | 12 (54.6) | 259 (51.4) | 0.93 | 12 (56.5) | 258 (51.3) | 0.27 | 0 (0.00) | 271 (51.6) | **0.05** |
| Vaginal IAP | 150 (24.7) | 17 (18.9) | 109 (25.0) |  | 4 (14.8) | 122 (24.5) |  | 14 (19.4) | 112 (24.7) |  | 4 (18.2) | 122 (24.2) |  | 3 (13.0) | 123 (24.5) |  | 0 (0.00) | 126 (24.0) |  |
| Elective CS IAP | 68 (11.2) | 13 (14.4) | 47 (10.8) |  | 4 (14.8) | 56 (11.2) |  | 8 (11.1) | 52 (11.5) |  | 3 (13.6) | 57 (11.3) |  | 5 (21.7) | 55 (10.9) |  | 1 (100.0) | 59 (11.2) |  |
| Emergency CS IAP | 81 (13.34) | 12 (13.3) | 57 (13.1) |  | 4 (14.8) | 65 (13.0) |  | 11 (15.3) | 58 (12.8) |  | 3 (13.6) | 66 (13.1) |  | 2 (8.7) | 67 (13.3) |  | 0 (0.00) | 69 (13.1) |  |
| Missing | 62 |  |  |  |  |  |  |  |  |  |  |  |  |  |  |  |  |  |  |
| Infant ethnicity |  |  |  |  |  |  |  |  |  |  |  |  |  |  |  |  |  |  |  |
| Asian | 77 (12.9) | 24 (26.7) | 39 (9.0) | **<0.01** | 12 (42.9) | 51 (10.3) | **<0.01** | 20 (27.8) | 43 (9.5) | **<0.01** | 9 (39.1) | 54 (10.8) | **<0.01** | 6 (26.1) | 57 (11.4) | **0.03** | 1 (100.0) | 62 (11.9) | 0.06 |
| First Nation | 47 (7.9) | 7 (7.8) | 31 (7.2) |  | 4 (14.3) | 34 (6.9) |  | 7 (9.7) | 31 (6.9) |  | 3 (13.0) | 35 (7.0) |  | 1 (4.4) | 37 (7.4) |  | 0 (0.0) | 38 (7.3) |  |
| Caucasian | 439 (73.5) | 55 (61.1) | 343 (79.2) |  | 11 (39.3) | 387 (78.2) |  | 43 (59.7) | 355 (78.1) |  | 11 (47.8) | 387 (77.4) |  | 13 (56.5) | 385 (77.0) |  | 0 (0.0) | 398 (76.3) |  |
| Other | 34 (5.7) | 4 (4.4) | 20 (4.6) |  | 1 (3.6) | 1 (3.6) |  | 2 (2.8) | 22 (4.9) |  | 0 (0.0) | 24 (4.8) |  | 3 (13.0) | 21 (4.2) |  | 0 (0.0) | 24 (4.6) |  |
| Missing | 72 |  |  |  |  |  |  |  |  |  |  |  |  |  |  |  |  |  |  |
| Season of birth |  |  |  |  |  |  |  |  |  |  |  |  |  |  |  |  |  |  |  |
| Summer (June-August) | 159 (26.0) | 21 (23.1) | 111 (25.3) | 0.66 | 4 (14.3) | 128 (25.5) | 0.18 | 16 (21.9) | 116 (25.4) | 0.53 | 3 (13.0) | 129 (25.4) | 0.18 | 6 (26.1) | 126 (24.9) | 0.89 | 0 (0.0) | 132 (25.0) | 0.56 |
| Other (October-May) | 453 (74.0) | 70 (76.9) | 328 (74.7) |  | 24 (85.7) | 374 (74.5) |  | 57 (78.1) | 341 (74.6) |  | 20 (87.0) | 378 (74.6) |  | 17 (73.9) | 381 (75.2) |  | 1 (100.0) | 397 (75.1) |  |
| Missing | 57 |  |  |  |  |  |  |  |  |  |  |  |  |  |  |  |  |  |  |
| Breastfeeding status at 3 months | |  |  |  |  |  |  |  |  |  |  |  |  |  |  |  |  |  |  |
| None | 93 (16.1) | 9 (10.1) | 70 (16.0) | 0.3 | 5 (17.9) | 74 (14.9) | 0.78 | 8 (11.3) | 71 (15.6) | 0.62 | 3 (13.0) | 76 (15.1) | 0.78 | 2 (8.7) | 77 (15.3) | 0.5 | 1 (100.0) | 78 (14.9) | 0.06 |
| Partial | 154 (26.7) | 23 (25.8) | 118 (27.0) |  | 6 (21.4) | 135 (27.1) |  | 19 (26.8) | 122 (26.8) |  | 5 (21.7) | 136 (27.0) |  | 5 (21.7) | 136 (27.0) |  | 0 (0.0) | 141 (26.9) |  |
| Exclusive | 329 (57.12) | 57 (64.0) | 249 (57.0) |  | 17 (60.7) | 289 (58.0) |  | 44 (62.0) | 262 (57.6) |  | 15 (65.2) | 291 (57.9) |  | 16 (69.6) | 290 (57.7) |  | 0 (0.0) | 306 (58.3) |  |
| Missing | 93 |  |  |  |  |  |  |  |  |  |  |  |  |  |  |  |  |  |  |
| Household income |  |  |  |  |  |  |  |  |  |  |  |  |  |  |  |  |  |  |  |
| <$50,000 | 73 (12.7) | 10 (11.2) | 51 (12.3) | 0.34 | 5 (18.5) | 56 (11.7) | 0.15 | 7 (9.9) | 54 (12.4) | 0.68 | 3 (13.6) | 58 (12.0) | 0.93 | 5 (21.7) | 56 (11.6) | **<0.01** | 1 (100.0) | 60 (11.9) | 0.06 |
| $50,000 to $99,000 | 192 (33.4) | 27 (30.4) | 146 (35.1) |  | 7 (25.9) | 166 (34.7) |  | 22 (31.0) | 151 (34.8) |  | 7 (31.8) | 166 (34.4) |  | 5 (21.7) | 168 (34.9) |  | 0 (0.0) | 173 (34.3) |  |
| ≥$100,000 | 271 (47.1) | 43 (48.3) | 197 (47.4) |  | 11 (40.7) | 229 (47.9) |  | 36 (50.7) | 204 (47.0) |  | 10 (45.5) | 230 (47.6) |  | 8 (34.8) | 232 (48.1) |  | 0 (0.0) | 240 (47.6) |  |
| Prefer not to say | 39 (6.8) | 9 (10.1) | 22 (5.3) |  | 4 (14.8) | 27 (5.7) |  | 6 (8.45) | 25 (5.8) |  | 2 (9.1) | 20 (6.0) |  | 5 (21.7) | 26 (5.4) |  | 0 (0.0) | 31 (6.2) |  |
| Missing | 94 |  |  |  |  |  |  |  |  |  |  |  |  |  |  |  |  |  |  |
| Maternal education |  |  |  |  |  |  |  |  |  |  |  |  |  |  |  |  |  |  |  |
| Highschool or less | 54 (9.4) | 6 (6.7) | 37 (8.9) | 0.3 | 0 (0.0) | 43 (9.0) | 0.43 | 4 (5.6) | 39 (9.0) | 0.15 | 0 (0.0) | 43 (8.9) | 0.4 | 2 (8.7) | 41 (8.5) | 0.32 | 0 (0.0) | 43 (8.5) | 0.63 |
| Some postsecondary | 210 (36.5) | 27 (30.3) | 157 (37.7) |  | 10 (37.0) | 174 (36.3) |  | 19 (26.8) | 165 (37.9) |  | 7 (31.8) | 177 (36.6) |  | 10 (43.5) | 174 (36.0) |  | 1 (100.0) | 183 (36.2) |  |
| University degree | 240 (41.7) | 46 (51.7) | 170 (40.8) |  | 13 (48.2) | 302 (42.4) |  | 38 (53.5) | 178 (40.9) |  | 11 (50.0) | 205 (42.4) |  | 11 (47.8) | 205 (42.4) |  | 0 (0.0) | 216 (42.8) |  |
| Postgraduate degree | 72 (12.5) | 10 (11.2) | 53 (12.7) |  | 4 (14.8) | 59 (12.3) |  | 10 (14.1) | 53 (12.2) |  | 4 (18.2) | 59 (12.2) |  | 0 (0.0) | 63 (13.0) |  | 0 (0.0) | 63 (12.5) |  |
| Missing | 93 |  |  |  |  |  |  |  |  |  |  |  |  |  |  |  |  |  |  |
| Maternal smoking |  |  |  |  |  |  |  |  |  |  |  |  |  |  |  |  |  |  |  |
| Yes | 24 (4.2) | 1 (1.1) | 17 (4.1) | 0.17 | 0 (0.0) | 18 (3.8) | 0.3 | 0 (0.0) | 18 (4.1) | 0.08 | 0 (0.0) | 18 (3.7) | 0.35 | 1 (4.4) | 17 (3.5) | 0.83 | 0 (0.0) | 18 (3.6) | 0.85 |
| No | 554 (95.9) | 89 (98.9) | 401 (95.9) |  | 28 (100.0) | 462 (96.3) |  | 72 (100.0) | 418 (95.9) |  | 23 (100.0) | 467 (96.3) |  | 22 (95.7) | 468 (96.5) |  | 1 (100.0) | 489 (96.5) |  |
| Missing | 91 |  |  |  |  |  |  |  |  |  |  |  |  |  |  |  |  |  |  |
| Maternal overweight/obesity | |  |  |  |  |  |  |  |  |  |  |  |  |  |  |  |  |  |  |
| Yes | 225 (43.4) | 35 (41.2) | 181 (43.2) | 0.73 | 7 (25.9) | 209 (43.8) | 0.07 | 29 (42.0) | 187 (43.0) | 0.88 | 7 (31.8) | 209 (43.4) | 0.29 | 6 (28.6) | 210 (43.5) | 0.18 | 0 (0.0) | 216 (42.9) | 0.39 |
| No | 294 (56.7) | 50 (58.8) | 238 (56.8) |  | 20 (74.1) | 268 (56.2) |  | 40 (58.0) | 248 (57.0) |  | 15 (68.2) | 273 (56.6) |  | 15 (71.4) | 273 (56.5) |  | 1 (100.0) | 287 (57.1) |  |
| Missing | 150 |  |  |  |  |  |  |  |  |  |  |  |  |  |  |  |  |  |  |
| Pets in home (pre or postnatal) | |  |  |  |  |  |  |  |  |  |  |  |  |  |  |  |  |  |  |
| Yes | 263 (54.7) | 33 (44.0) | 218 (58.8) | **0.02** | 10 (38.5) | 241 (57.4) | **0.06** | 23 (39.0) | 228 (58.9) | **<0.01** | 8 (38.1) | 243 (57.2) | 0.09 | 12 (57.1) | 239 (56.2) | 0.94 | 1 (100.0) | 250 (56.2) | 0.38 |
| No | 218 (45.3) | 41 (56.0) | 153 (41.2) |  | 16 (61.5) | 179 (42.6) |  | 36 (61.0) | 159 (41.1) |  | 13 (61.9) | 182 (42.8) |  | 9 (42.9) | 186 (43.8) |  | 0 (0.0) | 195 (43.8) |  |
| Missing | 188 |  |  |  |  |  |  |  |  |  |  |  |  |  |  |  |  |  |  |

CS, cesarean delivery; IAP, intrapartum antibiotics. p-value from Chi-squared test.
