# Supplementary material for: Natural Green Spaces, Sensitization to Allergens, and the Role of Gut Microbiota during Infancy
Source: mSystems. 2023 Feb 15;8(2):e01190-22. doi: 10.1128/msystems.01190-22 (PMC10134798; doi:10.1128/msystems.01190-22)
Supplement: TABLE S2 [file msystems.01190-22-s0002.docx]

|  |  | **Atopic sensitizations 3 years** | | | | | | **Food atopic sensitizations 3 years** | | | | | | **Inhalant atopic sensitizations 3 years** | | | | | |
| --- | --- | --- | --- | --- | --- | --- | --- | --- | --- | --- | --- | --- | --- | --- | --- | --- | --- | --- | --- |
|  |  | **≥1** | | | **≥2** | | | **≥1** | | | **≥2** | | | **≥1** | | | **≥2** | | |
|  | **Prevalence Overall  N (%)** | **Yes**  **N (%)** | **No**  **N (%)** | **p** | **Yes**  **N (%)** | **No**  **N (%)** | **p** | **Yes**  **N (%)** | **No**  **N (%)** | **p** | **Yes**  **N (%)** | **No**  **N (%)** | **p** | **Yes**  **N (%)** | **No**  **N (%)** | **p** | **Yes**  **N (%)** | **No**  **N (%)** | **p** |
| Total | 699 (100) | 75 (16.3) | 385 (83.7) |  | 31 (6.8) | 427 (93.2) |  | 38 (8.3) | 422 (91.7) |  | 10 (2.2) | 450 (97.8) |  | 58 (12.6) | 402 (87.4) |  | 15 (3.3) | 443 (96.7) |  |
| Infant sex |  |  |  |  |  |  |  |  |  |  |  |  |  |  |  |  |  |  |  |
| Male | 307 (50.3) | 46 (61.3) | 186 (48.3) | **0.04** | 22 (71.0) | 209 (48.9) | **0.02** | 23 (60.5) | 209 (49.5) | 0.19 | 8 (80.0) | 224 (49.8) | 0.06 | 38 (65.5) | 194 (48.3) | **0.01** | 11 (73.3) | 220 (49.7) | 0.07 |
| Female | 304 (49.8) | 29 (38.7) | 199 (51.7) |  | 9 (29.0) | 218 (51.1) |  | 15 (39.5) | 213 (50.5) |  | 2 (20.0) | 226 (50.2) |  | 20 (34.5) | 208 (51.7) |  | 4 (26.7) | 223 (50.3) |  |
| Missing | 58 |  |  |  |  |  |  |  |  |  |  |  |  |  |  |  |  |  |  |
| Birth weight (grams) | |  |  |  |  |  |  |  |  |  |  |  |  |  |  |  |  |  |  |
| <3,000 | 119 (19.6) | 22 (29.3) | 65 (17.0) | 0.09 | 6 (19.4) | 81 (19.1) | 0.87 | 8 (21.1) | 79 (18.9) | 0.68 | 3 (30.0) | 84 (18.8) | 0.25 | 69 (17.3) | 18 (31.0) | **0.05** | 4 (26.7) | 83 (18.9) | 0.49 |
| 3000-<3500 | 227 (37.3) | 23 (30.7) | 144 (37.7) |  | 11 (35.5) | 156 (36.8) |  | 16 (42.1) | 151 (36.0) |  | 2 (20.0) | 165 (36.9) |  | 152 (38.1) | 15 (25.9) |  | 6 (40.0) | 161 (36.6) |  |
| 3500-<4000 | 184 (30.3) | 22 (29.3) | 118 (30.9) |  | 11 (35.5) | 127 (30.0) |  | 11 (29.0) | 129 (30.8) |  | 5 (50.0) | 135 (30.2) |  | 121 (30.3) | 19 (32.8) |  | 2 (13.3) | 136 (30.9) |  |
| ≥4000 | 78 (12.8) | 8 (10.7) | 55 (14.4) |  | 3 (9.7) | 60 (14.1) |  | 3 (7.9) | 60 (14.3) |  | 0 (0.0) | 63 (14.1) |  | 57 (14.3) | 6 (10.3) |  | 3 (20.0) | 60 (13.6) |  |
| Missing | 61 |  |  |  |  |  |  |  |  |  |  |  |  |  |  |  |  |  |  |
| Gestational age (weeks) | |  |  |  |  |  |  |  |  |  |  |  |  |  |  |  |  |  |  |
| Preterm (34-36) | 34 (5.6) | 8 (10.7) | 20 (5.2) | 0.12 | 3 (9.7) | 25 (5.9) | 0.08 | 4 (10.5) | 24 (5.7) | 0.13 | 2 (20.0) | 26 (5.8) | 0.28 | 6 (10.3) | 22 (5.5) | 0.32 | 2 (13.3) | 26 (5.9) | **0.01** |
| Early term (37-38) | 145 (23.9) | 21 (28.0) | 80 (20.9) |  | 12 (38.7) | 89 (21.0) |  | 13 (34.2) | 88 (21.0) |  | 2 (20.0) | 99 (22.2) |  | 15 (25.9) | 86 (21.6) |  | 8 (53.3) | 93 (21.1) |  |
| Full term (39-40) | 339 (55.9) | 37 (49.3) | 221 (57.9) |  | 12 (38.7) | 245 (57.8) |  | 16 (42.1) | 242 (57.8) |  | 4 (40.0) | 254 (56.8) |  | 31 (53.5) | 227 (56.9) |  | 4 (26.7) | 253 (57.5) |  |
| Late term (≤41) | 89 (14.7) | 9 (12.0) | 61 (16.0) |  | 4 (12.9) | 65 (15.3) |  | 5 (13.2) | 65 (15.5) |  | 2 (20.0) | 68 (15.2) |  | 6 (10.3) | 64 (16.0) |  | 1 (6.7) | 68 (15.5) |  |
| Missing | 62 |  |  |  |  |  |  |  |  |  |  |  |  |  |  |  |  |  |  |
| Birth mode and IAP | |  |  |  |  |  |  |  |  |  |  |  |  |  |  |  |  |  |  |
| Vaginal, no IAP | 308 (50.7) | 35 (47.3) | 197 (51.4) | 0.18 | 15 (50.0) | 215 (50.6) | 0.97 | 20 (54.1) | 212 (50.5) | 0.89 | 7 (70.0) | 225 (50.3) | 0.38 | 25 (43.9) | 207 (51.8) | 0.08 | 8 (53.3) | 222 (50.5) | 0.96 |
| Vaginal IAP | 150 (24.7) | 16 (21.6) | 98 (25.6) |  | 7 (23.3) | 107 (25.2) |  | 10 (27.0) | 104 (24.8) |  | 1 (10.0) | 113 (25.3) |  | 12 (21.1) | 102 (25.5) |  | 3 (20.0) | 111 (25.2) |  |
| Elective CS IAP | 68 (11.2) | 7 (9.5) | 42 (11.0) |  | 3 (10.0) | 46 (10.8) |  | 3 (8.1) | 46 (11.0) |  | 0 (0.0) | 39 (11.0) |  | 6 (10.5) | 43 (10.8) |  | 2 (13.3) | 47 (10.7) |  |
| Emergency CS IAP | 81 (13.34) | 46 (12.0) | 46 (12.0) |  | 5 (16.7) | 57 (13.4) |  | 4 (10.8) | 58 (13.8) |  | 2 (20.0) | 60 (13.4) |  | 14 (24.6) | 48 (12.0) |  | 2 (13.3) | 60 (13.6) |  |
| Missing | 62 |  |  |  |  |  |  |  |  |  |  |  |  |  |  |  |  |  |  |
| Infant ethnicity | |  |  |  |  |  |  |  |  |  |  |  |  |  |  |  |  |  |  |
| Asian | 77 (12.9) | 16 (21.3) | 41 (10.7) | **0.01** | 9 (29.0) | 48 (11.3) | **<0.01** | 13 (34.2) | 44 (10.5) | **<0.01** | 3 (30.) | 54 (12.0) | **<0.01** | 11 (19.0) | 46 (11.5) | 0.37 | 4 (26.7) | 53 (12.0) | 0.06 |
| First Nation | 47 (7.9) | 9 (12.0) | 25 (6.5) |  | 6 (19.4) | 28 (6.6) |  | 6 (15.8) | 28 (6.7) |  | 4 (40.0) | 30 (6.7) |  | 5 (8.6) | 29 (7.2) |  | 3 (20.0) | 31 (7.0) |  |
| Caucasian | 439 (73.5) | 46 (61.3) | 302 (78.7) |  | 16 (51.6) | 331 (77.7) |  | 18 (47.4) | 330 (78.4) |  | 3 (30.0) | 345 (76.8) |  | 39 (67.2) | 309 (77.1) |  | 8 (53.3) | 339 (76.7) |  |
| Other | 34 (5.7) | 4 (5.3) | 16 (4.2) |  | 0 (0.0) | 19 (4.5) |  | 1 (2.6) | 19 (4.5)1 |  | 0 (0.0) | 20 (4.5) |  | 3 (5.2) | 17 (4.2) |  | 0 (0.0) | 19 (4.3) |  |
| Missing | 72 |  |  |  |  |  |  |  |  |  |  |  |  |  |  |  |  |  |  |
| Season of birth | |  |  |  |  |  |  |  |  |  |  |  |  |  |  |  |  |  |  |
| Summer (June-August) | 159 (26.0) | 11 (14.7) | 106 (27.5) | **0.02** | 4 (12.9) | 111 (26.0) | 0.11 | 6 (15.8) | 111 (26.3) | 0.15 | 1 (10.0) | 116 (25.8) | 0.26 | 9 (15.5) | 108 (26.9) | 0.06 | 3 (20.0) | 112 (25.3) | 0.64 |
| Other (October-May) | 453 (74.0) | 64 (85.3) | 279 (72.5) |  | 27 (87.1) | 316 (74.0) |  | 32 (84.2) | 311 (73.7) |  | 9 (90.0) | 334 (74.2) |  | 49 (84.5) | 294 (73.1) |  | 12 (80.0) | 331 (74.7) |  |
| Missing | 57 |  |  |  |  |  |  |  |  |  |  |  |  |  |  |  |  |  |  |
| Breastfeeding status at 3 months | |  |  |  |  |  |  |  |  |  |  |  |  |  |  |  |  |  |  |
| None | 93 (16.1) | 14 (19.2) | 51 (13.4) | 0.3 | 6 (20.0) | 59 (14.0) | 0.67 | 7 (18.9) | 58 (13.9) | 0.32 | 2 (20.0) | 63 (14.2) | 0.49 | 11 (19.3) | 54 (13.6) | 0.49 | 4 (28.6) | 61 (13.9) | **0.05** |
| Partial | 154 (26.7) | 15 920.6) | 103 (27.0) |  | 7 (23.3) | 111 (26.3) |  | 6 (16.2) | 112 (26.9) |  | 1 (10.0) | 117 (26.4) |  | 13 (22.8) | 105 (26.5) |  | 6 (42.9) | 112 (25.6) |  |
| Exclusive | 329 (57.12) | 44 (60.3) | 227 (59.6) |  | 17 (56.7) | 252 (59.7) |  | 24 (64.9) | 247 (59.2) |  | 7 (70.0) | 264 (59.5) |  | 33 (57.9) | 238 (60.0) |  | 4 (28.6) | 265 (60.5) |  |
| Missing | 93 |  |  |  |  |  |  |  |  |  |  |  |  |  |  |  |  |  |  |
| Household income | |  |  |  |  |  |  |  |  |  |  |  |  |  |  |  |  |  |  |
| <$50,000 | 73 (12.7) | 8 (11.4) | 38 (10.3) | 0.19 | 3 (10.0) | 43 (10.6) | 0.23 | 5 (13.9) | 41 (10.2) | 0.33 | 1 (11.11) | 45 (10.5) | 0.14 | 6 (10.9) | 40 (10.4) | 0.25 | 1 (6.7) | 45 (10.7) | 0.95 |
| $50,000 to $99,000 | 192 (33.4) | 20 (28.6) | 135 (36.6) |  | 9 (30.0) | 145 (35.6) |  | 8 (22.2) | 147 (36.5) |  | 2 (22.22) | 153 (25.6) |  | 18 (32.7) | 137 (35.7) |  | 5 (33.3) | 149 (35.3) |  |
| ≥$100,000 | 271 (47.1) | 35 (50.0) | 180 (48.8) |  | 14 (46.7) | 200 (49.1) |  | 20 (55.6) | 195 (48.4) |  | 4 (44.44) | 211 (49.1) |  | 25 (45.5) | 190 (49.5) |  | 8 (53.3) | 206 (48.8) |  |
| Prefer not to say | 39 (6.8) | 7 (10.0) | 16 (4.3) |  | 4 (13.3) | 19 (4.7) |  | 3 (8.3) | 20 (5.0) |  | 2 (22.22) | 21 (4.9) |  | 6 (10.9) | 17 (4.4) |  | 1 (6.67) | 22 (5.2) |  |
| Missing | 94 |  |  |  |  |  |  |  |  |  |  |  |  |  |  |  |  |  |  |
| Maternal education | |  |  |  |  |  |  |  |  |  |  |  |  |  |  |  |  |  |  |
| Highschool or less | 54 (9.4) | 1 (1.4) | 28 (7.6) | 0.11 | 1 (3.3) | 27 (6.6) | 0.53 | 1 (2.8) | 28 (6.9) | 0.27 | 0 (0.0) | 29 (6.7) | 0.85 | 1 (1.8) | 28 (7.3) | 0.29 | 1 (6.7) | 27 (6.4) | 0.9 |
| Some postsecondary | 210 (36.5) | 23 (32.4) | 129 (35.0) |  | 10 (33.3) | 142 (34.8) |  | 9 (25.0) | 143 (35.4) |  | 2 (22.2) | 149 (34.6) |  | 20 (35.7) | 132 (34.4) |  | 6 (40.0) | 146 (34.5) |  |
| University degree | 240 (41.7) | 40 (56.3) | 163 (44.2) |  | 17 (56.7) | 185 (45.3) |  | 22 (61.1) | 181 (44.8) |  | 5 (55.6) | 198 (45.9) |  | 30 (53.6) | 173 (45.1) |  | 7 (46.7) | 195 (46.1) |  |
| Postgraduate degree | 72 (12.5) | 7 (9.9) | 49 (13.3) |  | 2 (6.7) | 54 (13.2) |  | 4 (11.1) | 52 (12.9) |  | 1 (11.1) | 55 (12.8) |  | 5 (8.9) | 51 (13.3) |  | 1 (6.7) | 55 (13.0) |  |
| Missing | 93 |  |  |  |  |  |  |  |  |  |  |  |  |  |  |  |  |  |  |
| Maternal smoking | |  |  |  |  |  |  |  |  |  |  |  |  |  |  |  |  |  |  |
| Yes | 24 (4.2) | 1 (1.4) | 8 (2.2) | 0.66 | 1 (3.2) | 8 (2.0) | 0.63 | 0 (0.0) | 9 (2.2) | 0.36 | 0 (0.0) | 9 (2.1) | 0.65 | 1 (1.8) | 8 (2.1) | 0.88 | 1 (6.7) | 8 (1.9) | 0.2 |
| No | 554 (95.9) | 72 (98.6) | 363 (97.8) |  | 30 (96.8) | 403 (98.1) |  | 37 (100.0) | 398 (97.8) |  | 10 (100.0) | 425 (97.9) |  | 56 (98.3) | 379 (97.9) |  | 14 (93.3) | 419 (98.1) |  |
| Missing | 91 |  |  |  |  |  |  |  |  |  |  |  |  |  |  |  |  |  |  |
| Maternal overweight/obesity | |  |  |  |  |  |  |  |  |  |  |  |  |  |  |  |  |  |  |
| Yes | 225 (43.4) | 26 (37.7) | 162 (43.6) | 0.37 | 8 (27.6) | 179 (43.7) | 0.09 | 12 (35.3) | 176 (43.2) | 0.37 | 4 (40.0) | 184 (42.7) | 0.87 | 20 (36.4) | 168 (43.5) | 0.32 | 1 (7.1) | 186 (43.8) | **<0.01** |
| No | 294 (56.7) | 43 (62.3) | 210 (56.5) |  | 21 (72.4) | 231 (56.3) |  | 22 (64.7) | 231 (56.8) |  | 6 (60.0) | 247 (57.3) |  | 35 (63.6) | 218 (56.5) |  | 13 (92.9) | 239 (56.2) |  |
| Missing | 150 |  |  |  |  |  |  |  |  |  |  |  |  |  |  |  |  |  |  |
| Pets in home (pre or postnatal) | |  |  |  |  |  |  |  |  |  |  |  |  |  |  |  |  |  |  |
| Yes | 263 (54.7) | 33 (50.0) | 186 (56.9) | 0.31 | 10 (35.7) | 208 (57.1) | **0.03** | 12 (36.4) | 207 (57.5) | **0.02** | 2 (22.2) | 217 (56.5) | **0.04** | 28 (52.8) | 191 (56.2) | 0.65 | 5 (38.5) | 213 (56.2) | 0.21 |
| No | 218 (45.3) | 33 (50.0) | 141 (43.1) |  | 18 (64.3) | 156 (42.9) |  | 21 (63/6) | 153 (42.5) |  | 7 (77.8) | 167 (43.5) |  | 25 (47.2) | 149 (43.8) |  | 8 (61.5) | 166 (43.8) |  |
| Missing | 188 |  |  |  |  |  |  |  |  |  |  |  |  |  |  |  |  |  |  |

CS, cesarean delivery; IAP, intrapartum antibiotics. p-value from Chi-squared test.
