# Supplementary material for: Natural Green Spaces, Sensitization to Allergens, and the Role of Gut Microbiota during Infancy
Source: mSystems. 2023 Feb 15;8(2):e01190-22. doi: 10.1128/msystems.01190-22 (PMC10134798; doi:10.1128/msystems.01190-22)
Supplement: TABLE S3 [file msystems.01190-22-s0003.docx]

|  |  | **Odds of ≥1 Atopic Sensitization at 3 years** | | |  |  | **Odds of ≥2 Inhalant Atopic Sensitizations at 3 years** | | |  |
| --- | --- | --- | --- | --- | --- | --- | --- | --- | --- | --- |
|  |  | **N** | **Odds Ratio (95% CI)** | **p** |  |  | **N** | **Odds Ratio (95% CI)** | **p** |  |
| **Exposure to Natural Space <500m from Residence (Ref: No Natural Space)** | Crude Model | 460 | 1.75 (1.04, 2.95) | **0.03** | **Exposure to Natural Space <500m from Residence (Ref: No Natural Space)** | Crude Model | 458 | 0.38 (0.09, 0.90) | **0.03** |  |
|  | Missing Covariate Data Excluded Model | 392 | 1.87 (1.07, 3.28) | **0.03** |  | Missing Covariate Data Excluded Model | 391 | 0.34 (0.10, 1.13) | 0.08 |  |
|  | Adjusted for Covariates | 392 | 1.79 (0.99, 3.23) | 0.06 |  | Adjusted for Covariates | 391 | 0.30 (0.08, 1.04) | 0.06 |  |
