# Supplementary material for: Natural Green Spaces, Sensitization to Allergens, and the Role of Gut Microbiota during Infancy
Source: mSystems. 2023 Feb 15;8(2):e01190-22. doi: 10.1128/msystems.01190-22 (PMC10134798; doi:10.1128/msystems.01190-22)
Supplement: TABLE S4 [file msystems.01190-22-s0004.docx]

|  |  |  | **N** | **Odds Ratio (95% CI)** | **p** |
| --- | --- | --- | --- | --- | --- |
| **posure to Natural Space <500m from Residence (Ref: No Natural Space)** | **Atopic sensitizations 1 year** | **≥1** | 530 | 1.35 (0.85, 2.14) | 0.2 |
|  |  | **≥2** | 530 | 0.68 (0.32, 1.46) | 0.33 |
|  | **Atopic sensitizations 3 years** | **≥1** | 460 | 1.76 (1.04, 2.95) | **0.03** |
|  |  | **≥2** | 458 | 0.64 (0.31, 1.34) | 0.24 |
|  | **Food atopic sensitizations 1 years** | **≥1** | 530 | 1.34 (0.81, 2.23) | 0.25 |
|  |  | **≥2** | 530 | 0.73 (0.31, 1.68) | 0.45 |
|  | **Food atopic sensitizations 3 years** | **≥1** | 460 | 1.42 (0.71, 2.82) | 0.32 |
|  |  | **≥2** | 460 | 0.51 (0.23, 2.80) | 0.73 |
|  | **Inhalant atopic sensitizations 1 years** | **≥1** | 530 | 1.05 (0.45, 2.43) | 0.92 |
|  | **Inhalant atopic sensitizations 3 years** | **≥1** | 460 | 1.37 (0.78, 2.41) | 0.28 |
|  |  | **≥2** | 458 | 0.28 (0.09, 0.90) | **0.03** |
