# Supplementary figures and images for: Natural Green Spaces, Sensitization to Allergens, and the Role of Gut Microbiota during Infancy
Source: mSystems. 2023 Feb 15;8(2):e01190-22. doi: 10.1128/msystems.01190-22 (PMC10134798; doi:10.1128/msystems.01190-22)

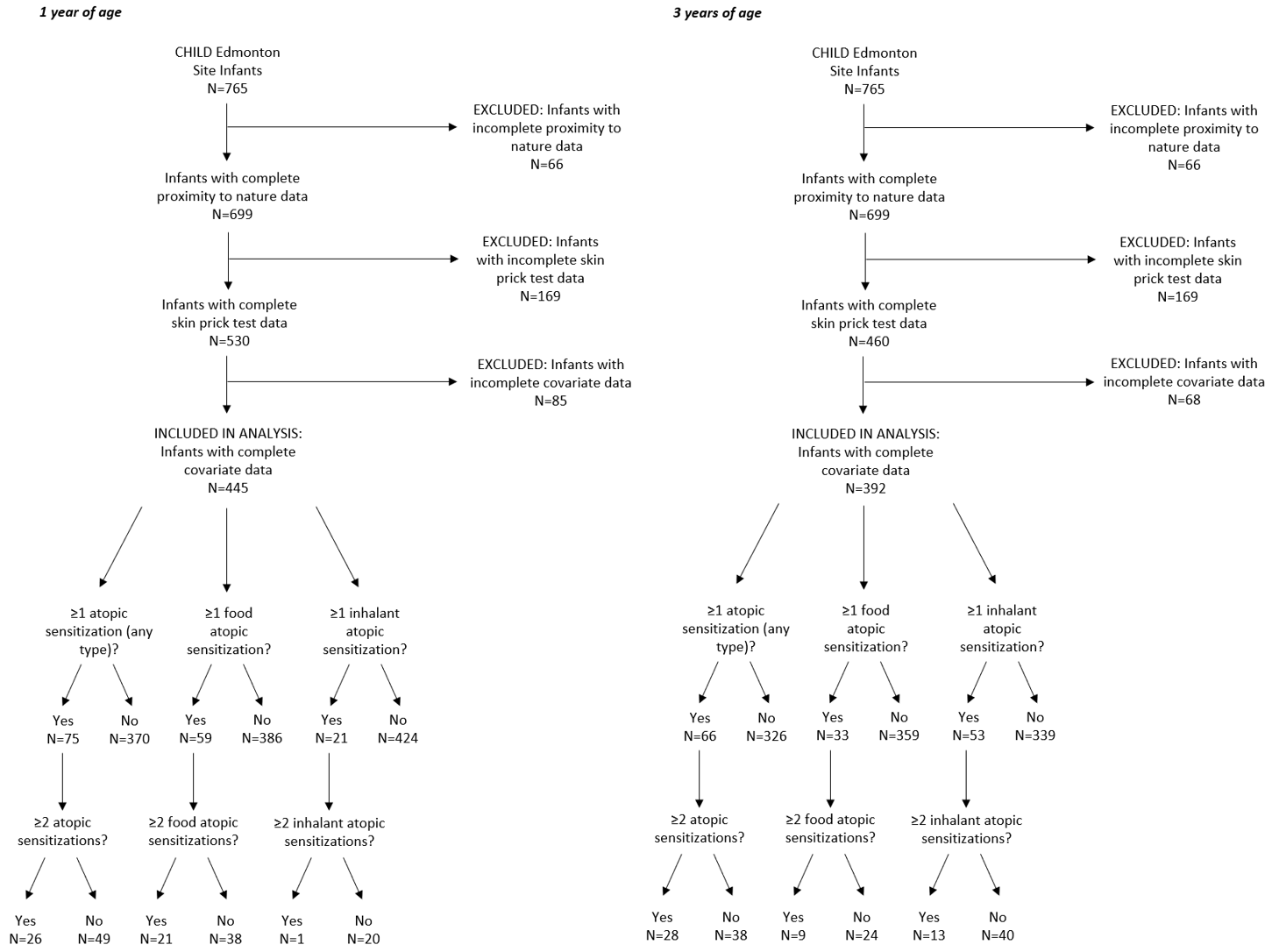

Supplement: FIG S1 [file msystems.01190-22-s0005.tif]

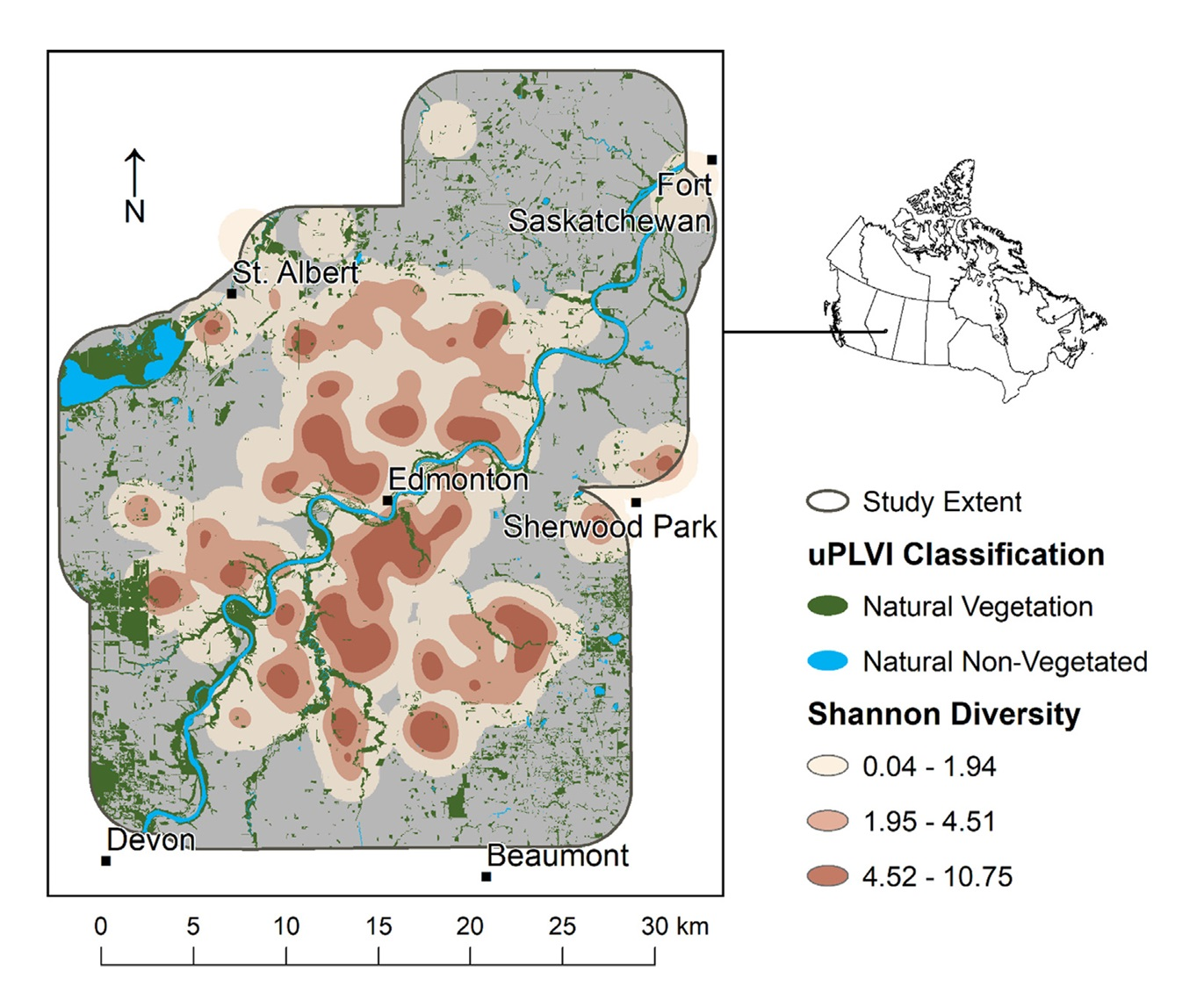

Supplement: FIG S2 [file msystems.01190-22-s0006.tif]

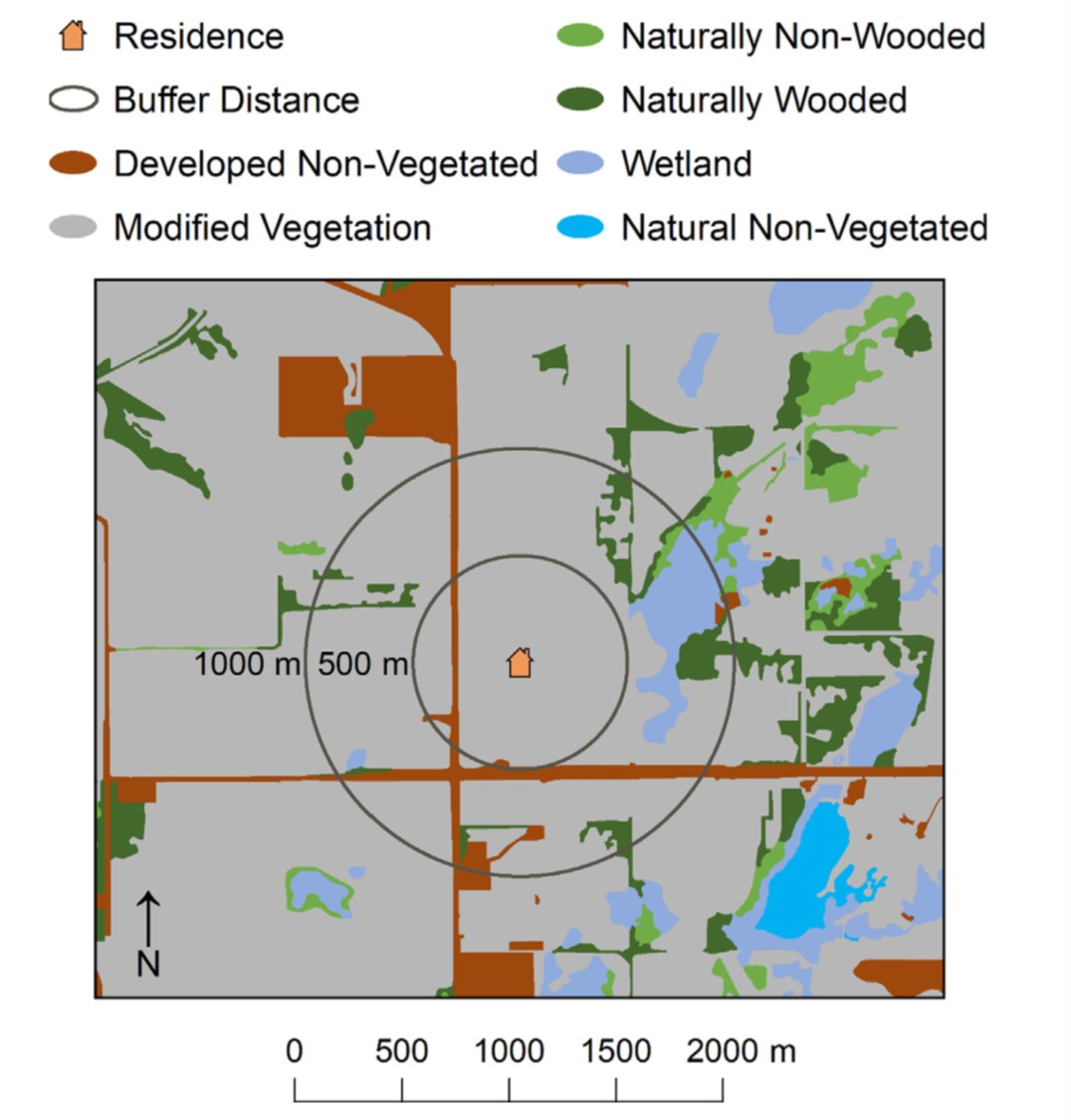

Supplement: FIG S3 [file msystems.01190-22-s0007.tif]

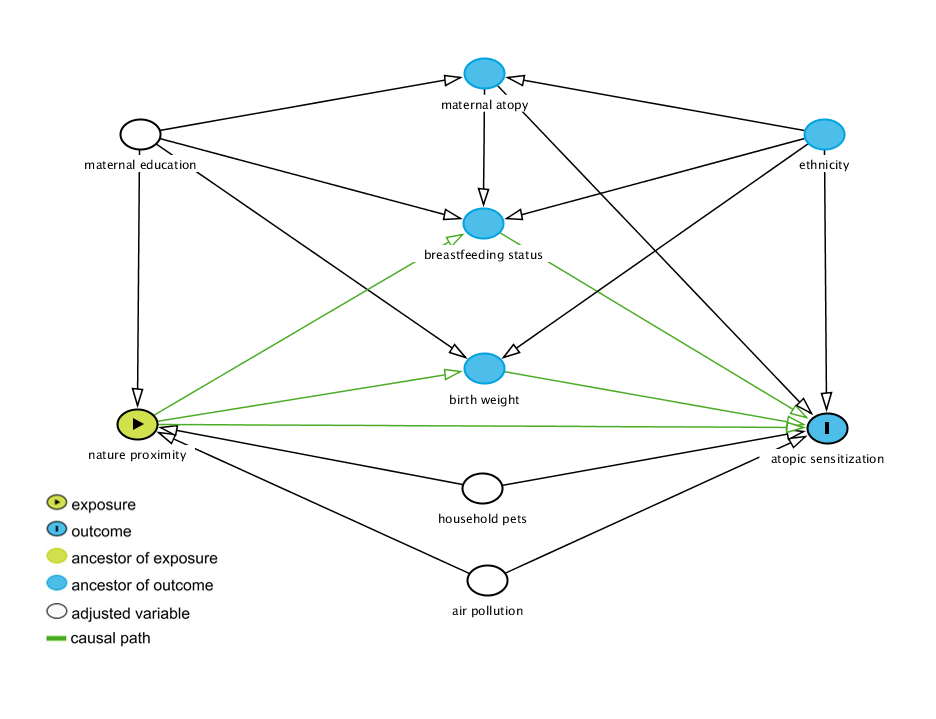

Supplement: FIG S4 [file msystems.01190-22-s0008.tif]
